# Identification of Methylated Gene Biomarkers in Patients with Alzheimer's Disease Based on Machine Learning

**DOI:** 10.1155/2020/8348147

**Published:** 2020-03-26

**Authors:** Jianting Ren, Bo Zhang, Dongfeng Wei, Zhanjun Zhang

**Affiliations:** ^1^State Key Laboratory of Cognitive Neuroscience and Learning & IDG/McGovern Institute for Brain Research, Beijing Normal University, Beijing 100875, China; ^2^BABRI Centre, Beijing Normal University, Beijing 100875, China; ^3^Clinical Laboratory, Xianghe Yuan Community Health Service Center, Beijing 100000, China; ^4^Institute of Basic Research in Clinical Medicine, China Academy of Chinese Medical Sciences, Beijing 100700, China

## Abstract

**Background:**

Alzheimer's disease (AD) is a neurodegenerative disorder and characterized by the cognitive impairments. It is essential to identify potential gene biomarkers for AD pathology.

**Methods:**

DNA methylation expression data of patients with AD were downloaded from the Gene Expression Omnibus (GEO) database. Differentially methylated sites were identified. The functional annotation analysis of corresponding genes in the differentially methylated sites was performed. The optimal diagnostic gene biomarkers for AD were identified by using random forest feature selection procedure. In addition, receiver operating characteristic (ROC) diagnostic analysis of differentially methylated genes was performed.

**Results:**

A total of 10 differentially methylated sites including 5 hypermethylated sites and 5 hypomethylated sites were identified in AD. There were a total of 8 genes including thioredoxin interacting protein (TXNIP), noggin (NOG), regulator of microtubule dynamics 2 (FAM82A1), myoneurin (MYNN), ankyrin repeat domain 34B (ANKRD34B), STAM-binding protein like 1, ALMalpha (STAMBPL1), cyclin-dependent kinase inhibitor 1C (CDKN1C), and coronin 2B (CORO2B) that correspond to 10 differentially methylated sites. The cell cycle (FDR = 0.0284087) and TGF-beta signaling pathway (FDR = 0.0380372) were the only two significantly enriched pathways of these genes. MYNN was selected as optimal diagnostic biomarker with great diagnostic value. The random forests model could effectively predict AD.

**Conclusion:**

Our study suggested that MYNN could be served as optimal diagnostic biomarker of AD. Cell cycle and TGF-beta signaling pathway may be associated with AD.

## 1. Introduction

Alzheimer's disease (AD), the most common form of neurodegenerative illness leading to dementia in elderly populations, affects approximately 32% of individuals over 85 and 11% of individuals over 65 years old [1]. By 2050, AD will affect as much as 1 in 85 people in the world [2]. AD is characterized with deposition of formation of neurofibrillary tangles, amyloid-*β* peptides as *β*-amyloid plaques, chronic neuroinflammation, and neuronal injury and loss [3]. It has been found that the dysfunction and death of neurons in brain regions, such as the amygdale, hippocampus, and cortical regions, contribute to the behavioral abnormalities in AD [4].

Clinically, AD is complex with multiple manifestations. AD etiology had the strong genetic component, with about 60–80% heritability [5]. The risk of genetic component of AD has been evidenced by the increased risk of AD among first-degree relatives of affected patients [6]. There are several other risk factors associated with AD, such as aging, age, activity, lifestyle, education, family history, and atherosclerosis [7]. Along with the progressively incapacitating, AD can linger many years. It is reported that AD can linger 8 years averagely, but it can also linger as long as 20 years [8]. Ultimately, AD is fatal and is estimated to be the leading cause of death [8]. In addition, the final diagnosis can only be got by autopsy making the identification of potential biomarkers of AD a great challenge [9]. Moreover, current treatments for AD are transient, not disease modifying. Therefore, it is needed to identify potential biomarkers for the diagnosis and therapy for AD.

DNA methylation is a crucial process in the regulation of gene expression in genetics. In AD genetics, the earliest genetic finding is the *ε*4 variant of the apolipoprotein E gene (APOE) [10]. In addition, the single-nucleotide polymorphism (rs11136000) of the clusterin (CLU) gene (encodes the protein similar to APOE) has also been associated with AD [11, 12]. In view of this, we tried to find the potential aberrant methylated genes in the pathology of AD based on the machine learning. We first obtained the DNA methylation data of patients with AD from the GEO database. Then, we performed the functional analyses of differentially methylated genes. Lastly, we applied machine learning to find the optimal diagnostic biomarker for AD.

## 2. Methods

### 2.1. Datasets Retrieval in the GEO Dataset

Herein, we searched datasets from the GEO dataset (http://www.ncbi.nlm.nih.gov/geo/) with the keywords “Alzheimer's disease”[MeSH Terms] OR Alzheimer's disease [All Fields] AND “Homo sapiens”[porgn] AND “gse”[Filter]. The study type was described as “Methylation profiling by array.” All selected datasets were genome-wide DNA methylation expression data of AD group and/or normal group superior temporal gyrus tissue samples. Only those standardized or primary datasets (the total samples size >50) were included. At last, a total of 2 datasets (GSE76105 and GSE59685) were identified, which was shown in Table 1.

### 2.2. Analysis of Differentially Methylated Sites in AD

Firstly, the primary data was preprocessed by an intersection taken of the two datasets, removing the sex chromosome sites and quantile standardization. Then, the COHCAP in the R package [13] was used to identify the differentially methylated sites. The threshold of differentially methylated sites was set as ∣Δbeta∣ > 0.2 and false discovery rate (FDR) < 0.05. Heat map of identified differentially methylated sites was generated by hierarchical clustering analysis by using R package.

### 2.3. Functional Annotation of Genes Corresponding to the Differentially Methylated Sites in AD

To investigate the biological function of genes corresponding to the differentially methylated sites, the online software GeneCodis3 (http://genecodis.cnb.csic.es/analysis) was applied to perform the functional annotation analysis of Gene Ontology (GO) classification and Kyoto Encyclopedia of Genes and Genomes (KEGG) pathway enrichment. The statistical significance was defined as FDR < 0.05.

### 2.4. Identify of the Optimal Diagnostic Gene Biomarkers for AD

To identify the optimal diagnostic gene biomarkers for AD, the feature selection procedures were performed as follows. Firstly, importance value of each differentially methylated site ranked according to the mean decrease in accuracy using the random forest algorithm. Then, the optimal number of features was found by subsequently adding one differentially methylated site at a time in a top-down forward-wrapper approach. Optimal differentially methylated sites with diagnostic value for AD were used to establish classification models including decision tree (DT), support vector machine (SVM) model, and random forests (RF). The “rpart” packet in R (https://cran.r-project.org/web/packages/rpart/), “e1071” package in R (https://cran.r-project.org/web/packages/e1071/index.html), and “randomForests” packet (https://cran.r-project.org/web/packages/randomForest/) establish the DT model, SVM model, and RF model, respectively. We compared three kinds of classification models by the average misjudgment rates of their 10-fold cross-validations. Diagnostic ability of classification prediction was evaluated by obtaining specificity, sensitivity, and the area under a receiver operating characteristic (ROC) curve (AUC).

### 2.5. Electronic Validation of Genes in Differentially Methylated Sites

The dataset of GSE63061 was used to validate the expression of genes in differentially methylated sites. It is noted that the GSE63061 dataset was comparable with the DNA methylation expression datasets of AD (GSE76105 and GSE59685) in terms of demographic and clinical characteristics (such as age, sex, and race). Clinical information statistics of the above 3 datasets was shown in supplementary [Supplementary-material supplementary-material-1]. The dataset of GSE63061 contains the blood sample of 139 patients with AD and 134 normal individuals. The expression result of these genes was visualized by box plots.

### 2.6. Diagnostic Analysis of Differentially Methylated Genes

By using pROC package in R language, we performed the receiver operating characteristic (ROC) analysis to assess the diagnostic value of differentially methylated genes. The area under the curve (AUC) under binomial exact confidence interval was calculated, and ROC curve was generated.

## 3. Results

### 3.1. Identification of Differentially Methylated Sites in AD

**Figure 1:**
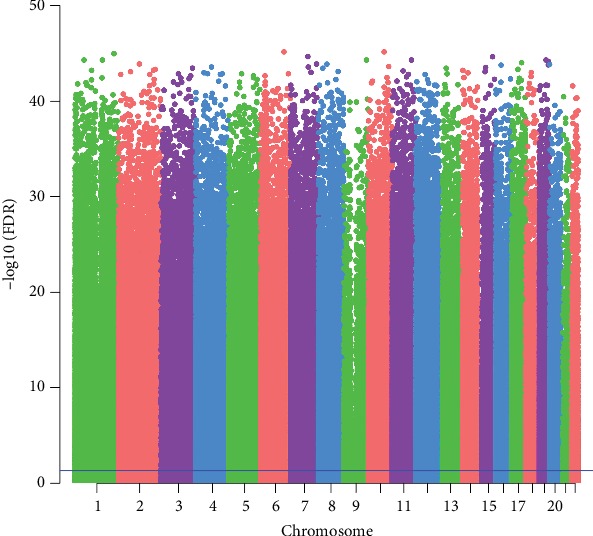
The Manhattan figure of 10 differentially methylated sites in AD. The *x*-axis represents the chromosome; the *y*-axis represents the -log10 (FDR) of differentially methylated sites.

**Figure 2:**
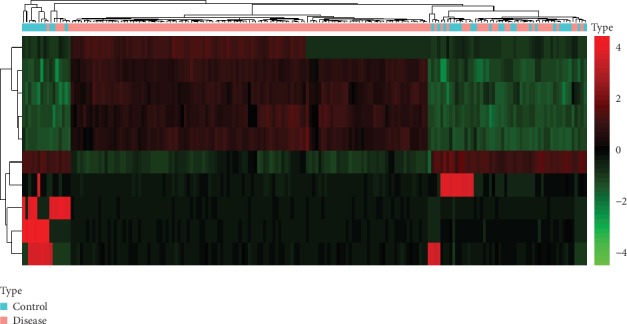
The heat map of 10 differentially methylated sites in AD. Diagram presents the result of a two-way hierarchical clustering of 10 differentially methylated sites and samples. The clustering is constructed using the complete-linkage method together with the Euclidean distance. Each row represents a differentially methylated site and each column, a sample. Differentially methylated sites clustering tree is shown in the bar on the right. The colour scale illustrates the relative level of differentially methylated site expression: red: below the reference channel; green: higher than the reference.

DNA methylation profiles of a total of 151 patients with AD and 34 normal individuals were obtained. After a series of data processing including intersection taken of two datasets, removing the sex chromosome sites and quantile standardization, a total of 438762 methylation sites were first detected. Then, a total of 10 differentially methylated sites including 5 hypermethylated sites and 5 hypomethylated sites were identified. Detailed information of 10 differentially methylated sites was presented in Table 2. The Manhattan figure of these differentially methylated sites was shown in Figure 1. The heat map of these differentially methylated sites was shown in Figure 2.

### 3.2. Functional Enrichment Analysis of Genes Corresponding to the Differentially Methylated Sites in AD

There were a total of 8 genes including thioredoxin interacting protein (TXNIP, hypermethylated), noggin (NOG, hypermethylated), regulator of microtubule dynamics 2 (FAM82A1, hypermethylated), myoneurin (MYNN, hypermethylated), ankyrin repeat domain 34B (ANKRD34B, hypermethylated), STAM-binding protein like 1, ALMalpha (STAMBPL1, hypomethylated), cyclin-dependent kinase inhibitor 1C (CDKN1C, hypomethylated), and coronin 2B (CORO2B, hypomethylated) that correspond to 10 differentially methylated sites. In order to investigate the potential biological function of these genes, GO and KEGG enrichment analysis were used for the functional analysis. GO enrichment analysis revealed that cellular response to tumor cell (FDR = 0.00283977), negative regulation of cytokine activity (FDR = 0.00283977), and positive regulation of glomerulus development (FDR = 0.00283977) were the most enriched biological processes; enzyme inhibitor activity (FDR = 0.0304319), protein kinase inhibitor activity (FDR = 0.0322006), and cytokine binding (FDR = 0.0357226) were the only enriched molecular functions; cytoplasm (FDR = 0.0463417) was the only enriched cellular component. KEGG enrichment analysis showed that the cell cycle (FDR = 0.0284087) and TGF-beta signaling pathway (FDR = 0.0380372) were the only enriched signal pathways. The result of enrichment analysis was showed in Table 3. It is a pity that one of the eight differentially methylated genes, STAMBPL1, was not involved in any biological process.

### 3.3. Identification of Optimal Diagnostic Gene Biomarkers for AD

**Figure 3:**
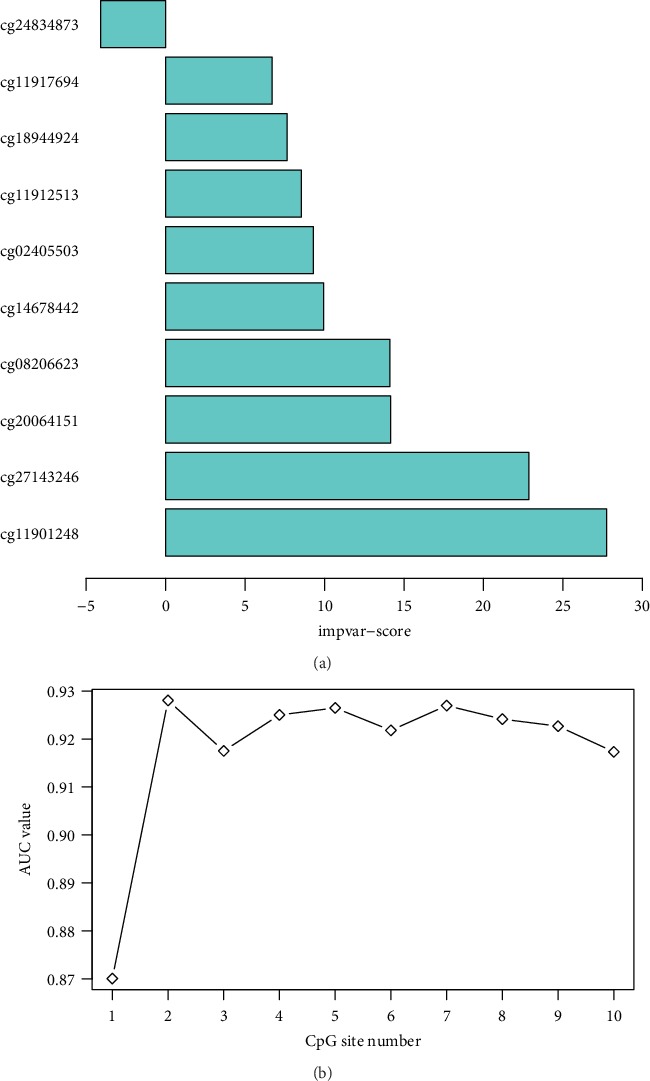
(a) The ranking of 10 differentially methylated sites. Differentially methylated sites were ranked according to the standardized drop in prediction accuracy. (b) The tendency chart of AUC along with the increase of methylation sites.

**Figure 4:**
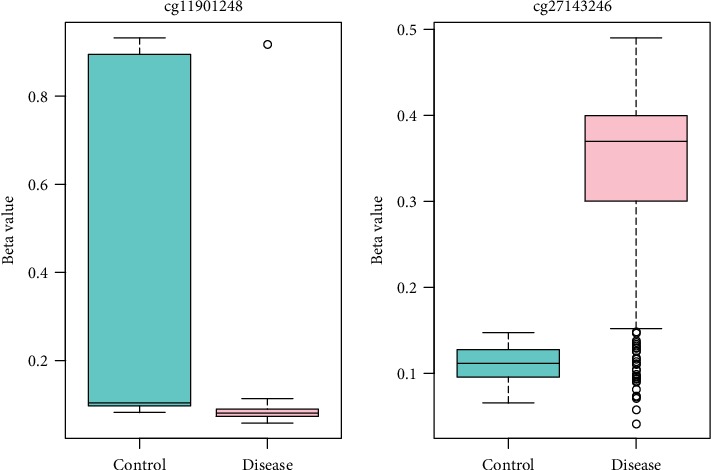
The box plots of the optimal differentially methylated sites for AD diagnose.

**Figure 5:**
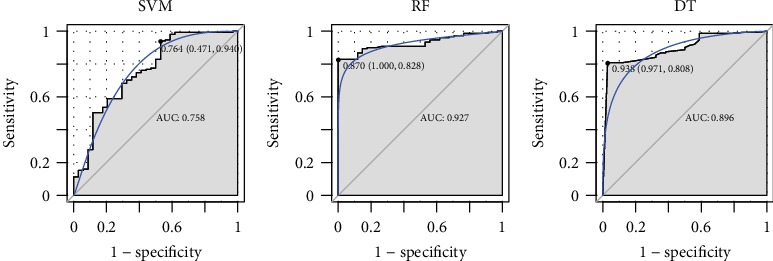
The ROC results of combinations of the two optimal diagnostic gene biomarkers based on DT, SVM, and RF classification model.

To identify the optimal diagnostic gene biomarkers for AD, the random forest feature selection and classification (DT, SVM, and RF) procedures were performed. All differentially methylated sites were ranked according to the standardized drop in prediction accuracy (Figure 3(a)). Differentially methylated sites including cg11901248 and cg27143246 were considered as the optimal diagnostic gene biomarkers for AD after subsequently adding one differentially methylated site at a time in a top-down forward-wrapper approach (Figure 3(b)). Box plots of the optimal differentially methylated sites in AD were presented in Figure 4. 2 optimal differentially methylated sites with diagnostic value for AD were used to establish classification models including DT, SVM, and RF. The 10-fold cross-validation indicated that the AUC value in the DT, SVM, and RF models was 89.6%, 75.8%, and 92.7%, respectively (Figure 5). It can be seen that the RF model is with the largest AUC value, which could effectively predict AD.

### 3.4. Electronic Validation of Genes in Differentially Methylated Sites

**Figure 6:**
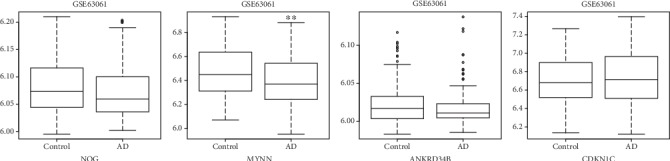
The box plots of electronic validation of NOG, MYNN, ANKRD34B, and CDKN1C in the GSE63061 dataset. ∗∗ represented *P* < 0.01.

In this study, 4 genes including NOG (hypermethylated), MYNN (hypermethylated), ANKRD34B (hypermethylated), and CDKN1C (hypomethylated) in differentially methylated sites were randomly selected for validation in the GSE63061 dataset (Figure 6). Our result showed that CDKN1C was up-regulated and that NOG, MYNN (*P* < 0.01), and ANKRD34B were down-regulated, which was consisted with the bioinformatics analysis.

### 3.5. Diagnosis Prediction of Differentially Methylated Genes

**Figure 7:**
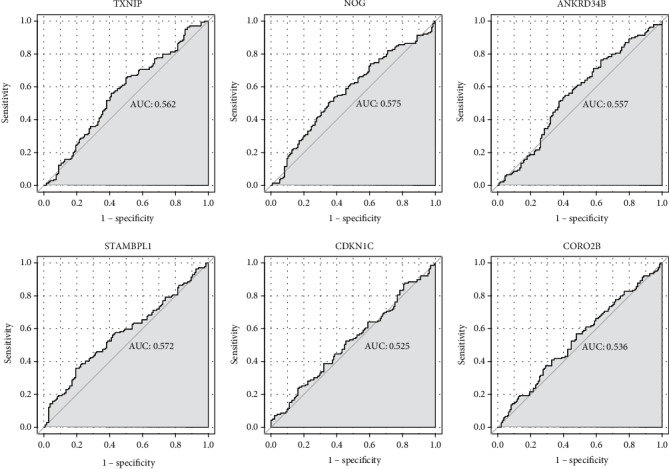
The ROC curves of TXNIP, NOG, ANKRD34B, STAMBPL1, CDKN1C, and CORO2B in AD. The ROC curves were used to show the diagnostic ability with 1-specificity and sensitivity.

ROC curve analysis was performed to assess the diagnosis ability of TXNIP, NOG, ANKRD34B, STAMBPL1, CDKN1C, and CORO2B (Figure 7). Unfortunately, AUC values of above differentially methylated genes were all <0.6, which suggested that they have no potential diagnostic value for AD.

## 4. Discussion

AD is a prevalent neurodegenerative disorder that severely affects the health of the old people. Therefore, exploring the potential biomarkers of AD is essential. In the present study, we performed integrated genome-wide analysis of DNA methylation expression profiles in patients with AD from GEO. A total of 10 differentially methylated sites including 5 hypermethylated sites and 5 hypomethylated sites were identified in AD. 10 differentially methylated sites were mapped to 8 genes including TXNIP, NOG, FAM82A1, MYNN, ANKRD34B, STAMBPL1, CDKN1C, and CORO2B. Among which, MYNN was served as optimal AD-specific diagnostic biomarker. The functional enrichment analysis showed that the cell cycle and TGF-beta signaling pathway were the only two significantly enriched pathways of these genes. The RF model could effectively predict AD.

TXNIP is an early response gene involved in neuronal apoptosis induced by high glucose and oxidative stress [14]. It mediates neuronal repair when transiently expressed [15, 16]. It is found that the expression of TXNIP is related to the senescence process and increases with age in the brain [17, 18]. It has been demonstrated that TXNIP is up-regulated in diabetes, ischemia, and hypertension, which were risk diseases for AD [15, 19–21]. Significantly, TXNIP is prominently increased in multiple brain regions including the superior frontal gyrus, postcentral gyrus, and entorhinal cortex in aging of AD [22]. In AD, the pharmacological inhibition of receptor for advanced glycation end product- (RAGE-) TXNIP axis will promote neuroprotection by blocking neurovascular dysfunction [23]. In addition, knockdown of hippocampal TXNIP can remarkably improve cognitive impairment and neuroinflammation, which suggested that TXNIP is a potential treatment target for AD [24]. In the present study, we first found the association between hypermethylated TXNIP and AD, which could provide new epigenetic evidence for AD pathology.

FAM82A1 (also called BLOCK18) is a potential and novel gene identified in human steroidogenesis and involved in the microtubule formation during cell division [25]. It is found that the expression of FAM82A1 is up-regulated in experimental autoimmune encephalomyelitis [26]. Up to now, there are few reports about the role of FAM82A1 in AD. Herein, we first found the association between hypermethylated FAM82A1 and AD, which suggested that FAM82A1 may be involved in AD.

The expression of ANKRD34B has been found in the brain of rodent [27]. It is reported that the CpG sites of ANKRD34B are significantly associated with age [28]. In mouse amyotrophic lateral sclerosis, the expression of ANKRD34B is down-regulated and plays roles in axon outgrowth and synapse formation in motor neurons [29]. In the peripheral blood of patients with bacterial meningitis, ANKRD34B is the most remarkably down-regulated gene [30]. In the present study, we found that ANKRD34B was hypermethylated in the tissue of AD, which was also validated in the blood sample of GSE63061 dataset. Our result suggested that ANKRD34B methylation may play key roles in the process of AD.

STAMBPL1 (also called AMSH-FP or AMSH-LP) is a member of the JAB1/MPN/MOV34 metalloenzyme (JAMM) family of zinc metalloproteases [31]. The expression of STAMBPL1 is increased in the middle cerebral artery [32]. Lavorgna and Harhaj found that STAMBPL1 regulated NF-*κ*B activation in neuroinflammation process [33]. It is worth mentioning that the missense mutation of STAMBPL1 has been found in AD in the Amish [7]. In this study, we also found the relationship between STAMBPL1 and AD, which further suggested that STAMBPL1 may be a crucial factor in the pathology of AD.

CORO2B, a central nervous system gene, is involved in brain cellular cytoskeleton rearrangement and motility and molecular trafficking [34, 35]. The up-regulated expression of CORO2B has been detected in induced neurons [36]. It is pointed out that CORO2B is associated with the neurological disease such as neuroblastoma [37]. In addition, CORO2B plays a key role in brain endothelial cells of cerebral malaria [38]. In the present study, we first found that CORO2B was hypomethylated in the tissues of AD, which indicated that CORO2B may be involved in the process of AD.

MYNN encodes the zinc-finger transcription factor myoneurin, which plays roles in regulating neuromuscular junctions [39]. Previous study has demonstrated the association between MYNN and AD [40]. Herein, we found that MYNN was hypermethylated in the tissues of AD. The expression tendency of MYNN was validated in the blood sample of GSE63061 dataset. Furthermore, it was identified as optimal diagnostic biomarker of AD by the method of machine learning. Our result may provide a new field in understanding the molecular mechanism and searching for the novel diagnostic biomarkers for AD.

According to the functional annotation of genes corresponding to the differentially methylated sites, we found that cell cycle and TGF-beta signaling pathway were the only two significant enrichment signaling pathways. Moreover, CDKN1C and NOF were the only genes that involved in the above two signaling pathways, respectively. Snape et al. reported that cell cycle defect was also one of the characteristics of AD [41]. The ectopic expression of several cell cycle proteins including p16, cdk4, PCNA, cyclin B1, and cdc2 kinase has been found in the brain regions of AD [42–44]. Moreover, it has been proposed that changes in these cell cycle proteins in lymphocytes can be considered as potential biomarkers for AD diagnosis [45–50]. CDKN1C (also called BWCR, BWS, KIP2, and WBS) is associated with neurogenesis and senescence [51, 52]. In the hippocampus, deletion of *CDKN1C* will increase neurogenesis, which leads to impaired neurogenesis [53]. It is noted that CDKN1C is up-regulated in severe AD [54]. Herein, we found that CDKN1C was hypomethylated both in the tissue and blood of AD. Furthermore, CDKN1C is also involved in the cell cycle, which suggested that CDKN1C may play a crucial role in AD.

Transforming growth factor-*β* (TGF-*β*), expressed by neurons, is a pleiotropic cytokine that regulates neuronal development and survival and protects neurons from central nervous system inflammation and injury [55–57]. Liao et al. found that the prompt and sustained expression change of TGF-*β* after brain injury may serve as the potential biomarker for brain injury [58]. Interestingly, it is reported that the TGF-*β* pathway is dysregulated in AD, and the accumulation around the amyloid plaques of TGF-*β* has been found in the brain of AD patients [59, 60]. The expression of TGF-*β* is up-regulated in brain tissue [61], while down-regulated in the serum of patients with AD [62]. NOG belongs to the transforming growth factor-*β* superfamily and is associated with neurorecovery and neuroregeneration [63]. Bonaguidi et al. and Yousef et al. found that NOG signaling changes with age and involved in the age-related neurological impairments and reductions in neuroregeneration [64, 65]. It is pointed that NOG is a pluripotent gene with increased expression in AD [66]. Herein, we found that NOG was hypermethylated in the tissues of AD. The electronic validation result in the blood sample was consisted with the informatics analysis in the tissues. Moreover, NOG was the only gene that involved in the TGF-*β* signaling pathway. Our results indicated that epigenetic change of NOG may be associated with AD pathology.

## 5. Conclusion

In summary, we found several differentially expressed methylated genes (TXNIP, NOG, FAM82A1, MYNN, ANKRD34B, STAMBPL1, CDKN1C, and CORO2B) in the tissues of AD. Importantly, MYNN may be the optimal diagnostic biomarker for AD. In addition, only two significantly enriched signaling pathways including cell cycle and TGF-*β* may provide a new field in understanding the pathological mechanism. However, there are limitations to our study. Firstly, some in vitro experiments such as quantitative real-time polymerase chain reaction, immumohistochemical staining, or western immunoblot are also needed to further validate the expression of identified genes. Secondly, we did not investigate the deeper mechanism of AD, and animal model or cell culture (A*β*-induced PC12 cells or primary neuron cells) is further needed to validate the expression of identified genes and explore the detailed function of identified methylated genes.

## Figures and Tables

**Table 1:** The DNA methylation expression datasets of AD.

GEO accession	Author	Platform	Samples (N:P)	Year	Country
GSE76105	Watson CT	Illumina HumanMethylation450 BeadChip	34 : 34	2015	USA
GSE59685	Lunnon K	Illumina HumanMethylation450 BeadChip	0 : 117	2017	UK

N: normal control; P: patients with AD.

**Table 2:** The detailed information of 10 differentially methylated sites in AD.

Site ID	Chr	Loc	Gene	Island	*Δ*beta	FDR
cg20064151	1	145438865	TXNIP	N/A	0.215096658	9.88741*E*-38
cg14678442	17	54672540	NOG	chr17:54674158-54674366	0.229358886	1.05581*E*-36
cg18944924	2	38265394	FAM82A1	N/A	0.209927033	3.71711*E*-39
cg27143246	3	169489583	MYNN	chr3:169490834-169491206	0.212332981	2.19602*E*-43
cg24834873	5	79865402	ANKRD34B	chr5:79864842-79866447	0.24222697	3.75651*E*-21
cg11917694	10	90639684	STAMBPL1	chr10:90639787-90640623	-0.20479899	0.020693382
cg08206623	11	2907334	CDKN1C	chr11:2907308-2907675	-0.21677658	9.7722*E*-40
cg02405503	15	68871738	CORO2B	chr15:68870633-68871974	-0.24286476	0.008382656
cg11901248	5	149866502	N/A	chr5:149865064-149866038	-0.26318932	0.003210476
cg11912513	9	43915234	N/A	chr9:43915270-43915506	-0.23938093	0.003403942

FDR: false discovery rate; N/A: not applicable.

**Table 3:** The result of GO and KEGG enrichment analysis.

Items	Items_Details	Support	FDR	Genes
Biological process				
GO:0071228	Cellular response to tumor cell	1	0.00283977	TXNIP
GO:0060302	Negative regulation of cytokine activity	1	0.00283977	NOG
GO:0090193	Positive regulation of glomerulus development	1	0.00283977	NOG
Molecular function				
GO:0004857	Enzyme inhibitor activity	1	0.0304319	TXNIP
GO:0004860	Protein kinase inhibitor activity	1	0.0322006	CDKN1C
GO:0019955	Cytokine binding	1	0.0357226	NOG
Cellular component				
GO:0005737	Cytoplasm	5	0.0463417	CORO2B,FAM82A1,ANKRD34B,CDKN1C,TXNIP
Signalling pathway				
Kegg:04110	Cell cycle	1	0.0284087	CDKN1C
Kegg:04350	TGF-beta signaling pathway	1	0.0380372	NOG

FDR: false discovery rate.

## Data Availability

All data are available in the manuscript.

## Supplementary Materials

Supplementary Materials Supplementary Table 1: clinical information statistics of above 3 datasets.
