# Supplementary material for: Identification of Methylated Gene Biomarkers in Patients with Alzheimer's Disease Based on Machine Learning
Source: Biomed Res Int. 2020 Mar 26;2020:8348147. doi: 10.1155/2020/8348147 (PMC7139879; doi:10.1155/2020/8348147)
Supplement: Supplementary Materials — Supplementary Table 1: clinical information statistics of above 3 datasets. [file 8348147.f1.doc]

|  | **Control**  **(n=168)** | **AD**  **(n=290)** | **Overall**  **(n=458)** | **P value** |
| --- | --- | --- | --- | --- |
| **Age** |  |  |  | 0.0958 |
| Mean (SD) | 76.4 (6.80) | 79.7 (7.17) | 78.4 (7.21) |  |
| Median [Min, Max] | 75.5 (63.0, 94.0) | 80.0 (59.0, 99.0) | 79.0 (59.0, 99.0) |  |
| Missing | 0 (0%) | 29 (10.0%) | 29 (6.3%) |  |
| **Gender** |  |  |  | 0.973 |
| Female | 98 (58.3%) | 171 (59.0%) | 269 (58.7%) |  |
| Male | 70 (41.7%) | 119 (41.0%) | 189 (41.3%) |  |
| **Race** |  |  |  | 0.061 |
| African America | 5 (3.0%) | 1 (0.3%) | 6 (1.3%) |  |
| Any other white background | 3 (1.8%) | 4 (1.4%) | 7 (1.5%) |  |
| Asian | 2 (1.2%) | 0 (0%) | 2 (0.4%) |  |
| British | 48 (28.6%) | 29 (10.0%) | 77 (16.8%) |  |
| Caucasian | 29 (17.3%) | 41 (14.1%) | 70 (15.3%) |  |
| Hispanic | 5 (3.0%) | 3 (1.0%) | 8 (1.7%) |  |
| Indian | 1 (0.6%) | 1 (0.3%) | 2 (0.4%) |  |
| Irish | 2 (1.2%) | 3 (1.0%) | 5 (1.1%) |  |
| Unknown | 0 (0%) | 118 (40.7%) | 118 (25.8%) |  |
| Western European | 72 (42.9%) | 90 (31.0%) | 162 (35.4%) |  |
| White and Asian | 1 (0.6%) | 0 (0%) | 1 (0.2%) |  |

**Supplementary Table 1 Clinical information statistics of above 3 dataset**
